# Beta-Blockers in the Management of Hypertension and/or Chronic Kidney Disease

**DOI:** 10.1155/2014/919256

**Published:** 2014-01-30

**Authors:** Hirofumi Tomiyama, Akira Yamashina

**Affiliations:** Second Department of Internal Medicine, Tokyo Medical University, 6-7-1 Nishi-Shinjuku, Shinjuku-ku, Tokyo 160-0023, Japan

## Abstract

This minireview provides current summaries of beta-blocker use in the management of hypertension and/or chronic kidney disease. Accumulated evidence suggests that atenolol is not sufficiently effective as a primary tool to treat hypertension. The less-than-adequate effect of beta-blockers in lowering the blood pressure and on vascular protection, and the unfavorable effects of these drugs, as compared to other antihypertensive agents, on the metabolic profile have been pointed out. On the other hand, in patients with chronic kidney disease, renin-angiotensin system blockers are the drugs of first choice for achieving the goal of renal protection. Recent studies have reported that vasodilatory beta-blockers have adequate antihypertensive efficacy and less harmful effects on the metabolic profile, and also exert beneficial effects on endothelial function and renal protection. However, there is still not sufficient evidence on the beneficial effects of the new beta-blockers.

## 1. Preface

Several recent meta-analyses have questioned the usefulness of beta-blockers as the primary tools to treat hypertension [1–3]. This has led to hesitation in the proper use of beta-blockers in the management of hypertension and chronic kidney disease (CKD). This review describes the opinion base promoting the use of beta-blockers for the treatment of hypertension and CKD.

## 2. Hypertension

### 2.1. Why Are Beta-Blockers Less Effective in the Prevention of Cardiovascular Events Than Other Antihypertensive Agents

Prichard classified beta-blockers into three types according to their beta1-selectivity and vasodilatory potential [4]. An additional classification is lipophilic or hydrophilic beta-blockers [5, 6]. Atenolol is a beta1-selective agent, and it has been widely used as the control drug in large randomized prospective controlled trials of newer antihypertensive agents such as calcium channel blockers and renin-angiotensin (RA) system blockers.  Table 1 summarizes the plausible reasons why beta-blockers are considered to be relatively ineffective for the prevention of cardiovascular events [7–9].

In the Anglo-Scandinavian Cardiac Outcomes Trial-Blood Pressure Lowering Arm (ASCOT-BPLA) study, blood pressure values were lower in those allocated to the calcium channel blocker-based regimen as compared to those allocated to the beta-blocker-based regimen throughout the trial period [10]. Recently, Webb et al. reported a meta-analysis in which they described visit-to-visit blood pressure instability in patients receiving beta-blocker treatment [11], and also that this instability was associated with an increased risk of stroke [12]. Atenolol was used in the ASCOT-BPLA study, and not only the analysis conducted by Webb et al. [11] but also that conducted by Rothwell et al. [12] involved the use of atenolol. Some studies demonstrated that once-daily atenolol does not provide adequate blood pressure control during the night-time and early morning periods because of its pharmacokinetic profile and half-life [13, 14]. These drug profiles of atenolol may be the cause for its relatively weak blood pressure-lowering effect and the blood pressure instability. On the other hand, metoprolol or bisoprolol have been shown to be more effective in sustaining 24-hour and early morning BP reductions as compared with atenolol [15, 16].

**Figure 1:**
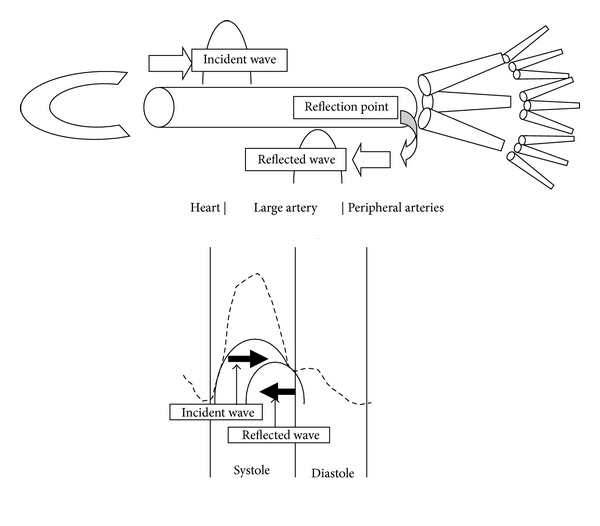
Schema of propagation of the incident pulse wave, reflected pulse wave, and their interaction in the arterial tree.

In the arterial tree, the arteries branch and taper as they reach peripheral sites, associated with the increase of the arterial resistance. Reflected pulse wave (from the periphery to the heart) occurs at sites of abrupt increase of the arterial resistance, such as at sites of arterial branching. Interaction between the incident pulse wave (from the heart to the periphery) and reflected pulse wave (from the periphery to the central region) is observed in the arterial tree (Figure 1); therefore, the blood pressure values differ between central and peripheral sites of the arterial tree [17]. Central (aortic and carotid) blood pressure is pathophysiologically more relevant than the peripheral pressure in the pathogenesis of cardiovascular disease [18]. Augmentation index (AI), a marker of the interaction of incident pressure wave and reflected pressure wave, was significantly and inversely related to heart rate due to an alteration in the relative timing of the reflected pressure wave [19]. Beta-blockers reduce the heart rate and decrease AI, which reduces their efficacy in reducing the central blood pressure as compared to other antihypertensive agents [20]. In their meta-analysis, Fagard et al. reported that beta-blockers exert a relatively weak effect in causing regression of the left ventricular mass [21]. In Fagard et al.'s review, atenolol was used in about 70% of the study subjects prescribed beta-blockers, and no study involving the use of vasodilatory beta-blockers was included. Recently, the advantages of nebivolol, a vasodilatory beta-blocker, over conventional *β*-blockers in reducing the central blood pressure and inducing regression of the left ventricular mass have been reported [22]. Compared with atenolol, nebivolol exerts a more favorable effect on 24-hour blood pressure profile [23]. Furthermore, nebivolol and telmisartan, an angiotensin II receptor blocker, decreased the left ventricular mass to a similar degree [24].

Shahin et al. reported that angiotensin-converting enzyme inhibitors improve endothelial function and are superior antihypertensive agents as compared to calcium channel blockers and beta-blockers [25]. However, in all of the studies cited in their meta-analysis, atenolol had been used as the beta-blocker. In contrast to atenolol, carvedilol and nebivolol also improve the endothelial function [26, 27].

While the meta-analysis conducted by Messerli et al. reported the unfavorable effects of beta-blockers on the metabolic profiles, this analysis did not include studies in which vasodilatory beta-blockers had been used [28]. More recent studies have reported the relatively less harmful effects of vasodilatory beta-blockers on the metabolic profiles and also on weight gain [29, 30].

As described above, new generations of beta-blockers, such as the long-acting and/or vasodilatory beta-blockers may overcome the relatively weak effect of beta-blockers in preventing cardiovascular events.

### 2.2. Concerns about Recent Meta-Analyses

A Cochrane Collaboration analysis conducted by Wiysonge et al., which was a representative analysis to evaluate the usefulness of beta-blockers in the management of hypertension, suggested that first-line beta-blocker use was not as good as other classes of antihypertensive drugs to decrease the mortality or morbidity [1, 2]. In his review, 60–70% of the subjects were receiving atenolol. As mentioned above, there is some doubt about the suitability of atenolol as a first-line antihypertensive drug, because of its low lipophilic profile and relatively weak effect on cardiovascular protection [5].

The MAPHY study demonstrated the significantly lower risk for coronary events in patients on metoprolol, a lipophilic beta-blocker, as compared to those on diuretics [31]. The usefulness of lipophilic beta-blockers for the prevention of cardiovascular events is still under debate [5, 6]. The meta-analysis conducted by Wiysonge et al. (total number of analyzed subjects, 91561) reported the higher risk for cardiovascular events in patients on beta-blockers as compared to those on diuretics. However, the number of study subjects prescribed metoprolol included in their meta-analysis was 7663 (8.4%). On the other hand, the meta-analysis conducted by Turnbull et al. (total number of study subjects for the comparison of the outcomes of major cardiovascular events (angiotensin converting enzyme inhibitor or calcium antagonist versus beta-blocker) was 14583, which was calculated from Figure 4 in [32]) demonstrated no evidence of any difference in the effect between beta-blockers and other classes of antihypertensive agents in preventing major cardiovascular events [32]. This meta-analysis included two studies in which metoprolol alone was used in the beta-blocker arm and two other studies in which metoprolol was used as one of the beta-blockers in the beta-blocker arm. The number of study subjects prescribed metoprolol included in this meta-analysis was 10062 (13.5%). Thus, the meta-analysis conducted by Turnbull et al. might have included a lower number of subjects prescribed atenolol and higher number of study subjects prescribed metoprolol, as compared to the meta-analysis conducted by Wiysonge et al. [1]. Then, recently, Turnbull et al. suggested that lipophilic beta-blockers may be preferable to hydrophilic beta-blockers for reducing the mortality in patients with coronary artery disease [33], though lipophilic *β*-blockers are associated with an increased risk of depressive symptoms [34].

Lindholm et al. reported that the differential effects between nonatenolol beta-blockers and other antihypertensive drugs on the risk of major cardiovascular events could not be fully evaluated because of the small number of studies including subjects prescribed nonatenolol beta-blockers [3]. Anyhow, atenolol is one of the most widely used beta-blockers, and more than 50% of the data in previous meta-analyses were derived from subjects prescribed atenolol. A meta-analysis to examine the effects of lipophilic and/or vasodilatory beta-blockers on the risk of major cardiovascular events is proposed.

### 2.3. Heart Rate in Beta-Blockers

**Figure 2:**
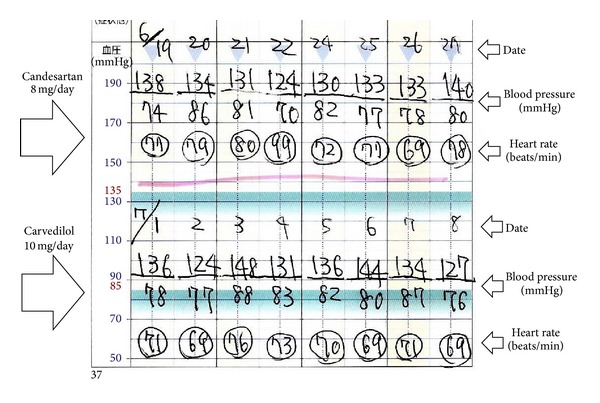
Self-measured blood pressure and heart rate at home under treatment with candesartan and under treatment with carvedilol.

The meta-analysis conducted by Bangalore et al. demonstrated an inverse relationship between beta-blocker-induced heart rate lowering and the reduction in the risk of future cardiovascular events in patients with hypertension [35]. This heart rate lowering causes a pseudoantihypertensive effect; that is, the central aortic pressure becomes less than the brachial pressure [7, 17, 18]. This phenomenon is thought to be one of mechanisms underlying the lower cardiovascular-protective effects of beta-blockers. On the other hand, high heart rate is known as an independent risk factor for major cardiovascular events. The risk seems to increase as the heart rate begins to exceed 70 bpm [36]. The aforementioned meta-analysis conducted by Bangalore et al. demonstrated an inverse relationship between heart rate and cardiovascular events in subjects with heart rates under 70 bpm, and no study until the current date has examined the relationship between the heart rate and the risk for cardiovascular events in cases receiving beta-blocker treatment with heart rates over 70 bpm. Furthermore, atenolol was used in more than 80% of the study subjects of the meta-analysis conducted by Bangalore et al. [35]. Thus, no study has examined the association between the reduction in heart rate and the increased risk of cardiovascular events by the treatment with beta-blockers other than atenolol. Our previous prospective study identified high heart rate as an independent risk factor for vascular damage (increase in arterial stiffening) [37]. Therefore, it has not been concluded that beta-blocker-induced heart rate lowering increases the risk of cardiovascular events in cases with heart rate over 70 bpm. In the case of patients with hypertension and a high heart rate, we have sometimes experienced good efficacy of beta-blockers for the control of both the blood pressure and the heart rate (Figure 2).

## 3. CKD

### 3.1. Renal Protection and Beta-Blockers in CKD

In addition to the prevention of cardiovascular events, renal protection is crucial in the management of CKD. Recent guidelines recommended RA system blockers as the agents of first choice for the management of hypertension in patients with CKD, because of the significant renal-protective effects of this class of drugs [38, 39]. On the other hand, the sympathetic nervous system is activated in CKD [40, 41], which acts as a key player in the progression of renal dysfunction and may also contribute to the onset/progression of cardiovascular disease [40, 41]. However, reduction of the cardiac output and the consequent impairment of renal perfusion caused by beta-blockers are thought to be harmful in patients with CKD [42]. Actually, recent studies have demonstrated that only 20–30% of patients with CKD are prescribed beta-blockers [43, 44].

The Kidney Disease Outcomes Quality Initiative (K/DOQI) guideline recommend that beta-blockers be used as the third-line antihypertensive agents in patients with proteinuria [45]. However, as mentioned above, while meta-analyses have suggested the demerits of atenolol in the management of hypertension [1–3], controversial results of the renal-protective effects of atenolol as compared to those of other antihypertensive agents were reported in subjects with hypertension accompanying CKD [46, 47]. Concerning other beta-blockers, the African American Study of Kidney Disease and Hypertension Study (AASK Trials) demonstrated the absence of any significant differences in the clinical composite outcomes (renal function decline, onset of end-stage renal disease, and/or death) between patients treated with metoprolol and those treated with amlodipine [48]. Two multicenter studies reported the renal protective effects of carvedilol, a vasodilatatory beta-blocker [29, 49]. Thus, it has not yet been concluded whether beta-blockers (especially vasodilatory beta-blockers) may simply represent third-line of antihypertensive agents for the management of hypertension in patients with CKD. Further study is proposed to clarify whether calcium channel blockers (especially nondihydropyridines, which reduce proteinuria) [50] or vasodilatory beta-blockers may be more suitable for renal protection and controlling blood pressure in patients with CKD.

### 3.2. Prevention of Cardiovascular Events, including Sudden Death, and Beta-Blockers in CKD

Traditional risk factors such as hypertension, diabetes mellitus, and dyslipidemia, and nontraditional risk factors such as inflammation, oxidative stress, abnormal mineral metabolisms, hyperparathyroidism, homocysteinemia, and anemia are known to be associated with cardiovascular events in patients with CKD [51]. The reported rate of sudden cardiac death in patients with end-stage renal disease is 50-fold higher than that in the general population [52]. In addition to the prevalence of coronary artery disease and/or heart failure, left ventricular hypertrophy, electrolyte abnormalities such as hyperkalemia, and vascular calcification might also be associated with sudden cardiac death in patients with CKD [52].

Badve et al.'s meta-analysis demonstrated that beta-blockers reduce the risk of all-cause and cardiovascular mortality in patients with CKD and systolic heart failure [53]. Basically, in their review, studies involving the use of beta-blockers other than atenolol were included, and ACE inhibitors were concomitantly prescribed for most of the patients in these studies. However, their meta-analysis did not evaluate the effect of beta-blockers on sudden cardiac death. The hemodialysis (HEMO) study suggested a trend towards the benefit of beta-blockers for the prevention of sudden cardiac death in patients with CKD and coronary heart disease, but not in CKD patients without coronary artery disease (this study did not describe the details about the kind of beta-blockers used, e.g., atenolol) [54].

## 4. Summary

### 4.1. Hypertension

This review does not support the use of beta-blockers as the primary tool to treat hypertension. However, it does propose proper use of beta-blockers, especially in cases with high heart rate and/or resistant hypertension, considering their long-acting, vasodilatory, and/or lipophilic profiles.

### 4.2. CKD

Beta-blockers are recommended as second-line agents after RA system blockers for controlling hypertension in patients with CKD and systolic heart failure. As compared to other antihypertensive agents, except RA system blockers, it has been confirmed that there are no demerits to using beta-blockers for renal protection. In addition, vasodilatory beta-blockers may also have beneficial renal-protective effects. Even in patients with CKD, control of blood pressure is crucial for the prevention of cardiovascular events. While the combination of RA system blockers with diuretics is effective for reducing the blood pressure, in CKD often three or more different antihypertensive drugs are required to control blood pressure level. Then, there is no evidence that beta-blockers, especially vasodilatory beta blockers, are inferior to diuretics or calcium channel blockers as second- or third-line agents for renal protection and control of blood pressure in patients with CKD [55].

## Figures and Tables

**Table 1:** Plausible reasons for beta-blockers being relatively ineffective for the prevention of cardiovascular events.

Less effective lowering of the blood pressure
Visit-to-visit blood pressure instability
Less effective lowering of the central blood pressure
Less effective regression of the left ventricular hypertrophy
Unfavorable metabolic effects
Less effective vascular protection
Reduced drug compliance
